# Inhibition on α-Glucosidase Activity and Non-Enzymatic Glycation by an Anti-Oxidative Proteoglycan from *Ganoderma lucidum*

**DOI:** 10.3390/molecules27051457

**Published:** 2022-02-22

**Authors:** Ying Zhang, Yanna Pan, Jiaqi Li, Zeng Zhang, Yanming He, Hongjie Yang, Ping Zhou

**Affiliations:** 1 State Key Laboratory of Molecular Engineering of Polymers, Department of Macromolecular Science, Fudan University, Shanghai 200433, China; 19210440005@fudan.edu.cn (Y.Z.); 18110440051@fudan.edu.cn (Y.P.); 20210440013@fudan.edu.cn (J.L.); 2 Yueyang Hospital of Integrated Traditional Chinese and Western Medicine, Shanghai University of Traditional Chinese Medicine, Shanghai 200437, China; zengzeng31@163.com (Z.Z.); heyanming176@163.com (Y.H.)

**Keywords:** *Ganoderma lucidum*, postprandial hyperglycemia, α-glucosidase, non-enzymatic glycation, advanced glycation end products (AGEs)

## Abstract

The prevention of postprandial hyperglycemia and diabetic complications is crucial for diabetes management. Inhibition of α-glucosidase to slow carbohydrate metabolism is a strategy to alleviate postprandial hyperglycemia. In addition, suppression of non-enzymatic glycation can diminish the advanced glycation end products and reduce the oxidative stress and inflammation, thereby preventing the diabetic complications. In this study, an anti-oxidative proteoglycan (named *FYGL*) extracted from *Ganoderma lucidum* was investigated in vitro for its inhibitory effect on α-glucosidase and non-enzymatic glycation using molecular kinetics, intrinsic fluorescence assay, and bovine serum albumin glycation models. The molecular kinetics and fluorescence assay revealed that *FYGL* decreases α-glucosidase activity by forming a *FYGL*–α-glucosidase complex. To evaluate the anti-glycation effect, fructose-glycated and methylglyoxal-glycated BSA models were analyzed by spectroscopic and SDS-PAGE methods. Results showed that *FYGL* inhibited the glycation at every stage and suppressed glycoxidation, possibly due to its anti-oxidative capacity and *FYGL*–BSA complex formation. Furthermore, we demonstrated in vivo that *FYGL* could alleviate postprandial hyperglycemia in *db/db* mice as well as AGE accumulation and vascular injury in diabetic rats. Overall, *FYGL* possesses anti-postprandial hyperglycemia and anti-glycation functions and would be potentially used in clinic for diabetes and related complication management.

## 1. Introduction

Diabetes is a common metabolic disease characterized by hyperglycemia. Postprandial blood glucose (PBG) and fasting blood glucose (FBG) are two major indicators of hyperglycemia. High postprandial blood glucose accelerates the occurrence of microvascular and macrovascular complications as well as type II diabetes [1]. Strict control of PBG is very important for the prevention and management of diabetes mellitus [2]. After meals, postprandial blood glucose reaches its peak due to the digestion of carbohydrates such as starch and sucrose in food. Starch is decomposed into oligosaccharides or disaccharides by α-amylase in the saliva and pancreatic juice and then further decomposed into absorbable glucose by α-glucosidase at the brush edge of small intestinal epithelial cells, leading to the PBG peak reached. Inhibiting the α-glucosidase activity can alleviate postprandial hyperglycemia [3]; therefore, finding efficient α-glucosidase inhibitors (AGIs) has gained much attention [4]. In addition, continuous hyperglycemia in diabetic patients accelerates non-enzymatic glycation and increases the production of advanced glycation end products (AGEs) [5]. AGEs are formed in the extracellular matrix (ECM), such as elastin and lipids, resulting in the denaturation of its constitution and the stiffness of the matrix. Furthermore, AGEs interact with their receptor on vascular endothelial cell surfaces, leading to the activation of the pathway of AGE and its receptor (RAGE) and oxidative stress and inflammation [6], which accelerate diabetic complications, especially vascular complications [7]. Therefore, inhibiting AGE formation is particularly important to protect against diabetic vascular injury and other complications [8].

The drugs acarbose, miglitol, and voglibose have been clinically proven to control postprandial hyperglycemia by inhibiting α-glucosidase activity [9]. However, these AGIs have gastrointestinal side effects to some extent, such as abdominal distention, intestinal distress, abdominal pain, and diarrhea [10]. Furthermore, aminoguanidine (AG), a synthetic AGE inhibitor, has been forbidden in clinic due to its adverse effects, such as anemia and gastrointestinal disturbances [11]. In recent years, natural products have been found to be safe and have been given more attention to alleviate postprandial hyperglycemia and non-enzymatic glycation [12,13].

**Figure 1:**
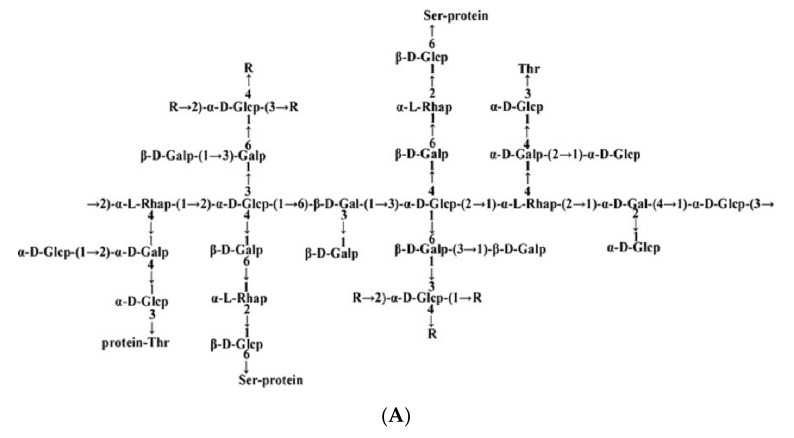

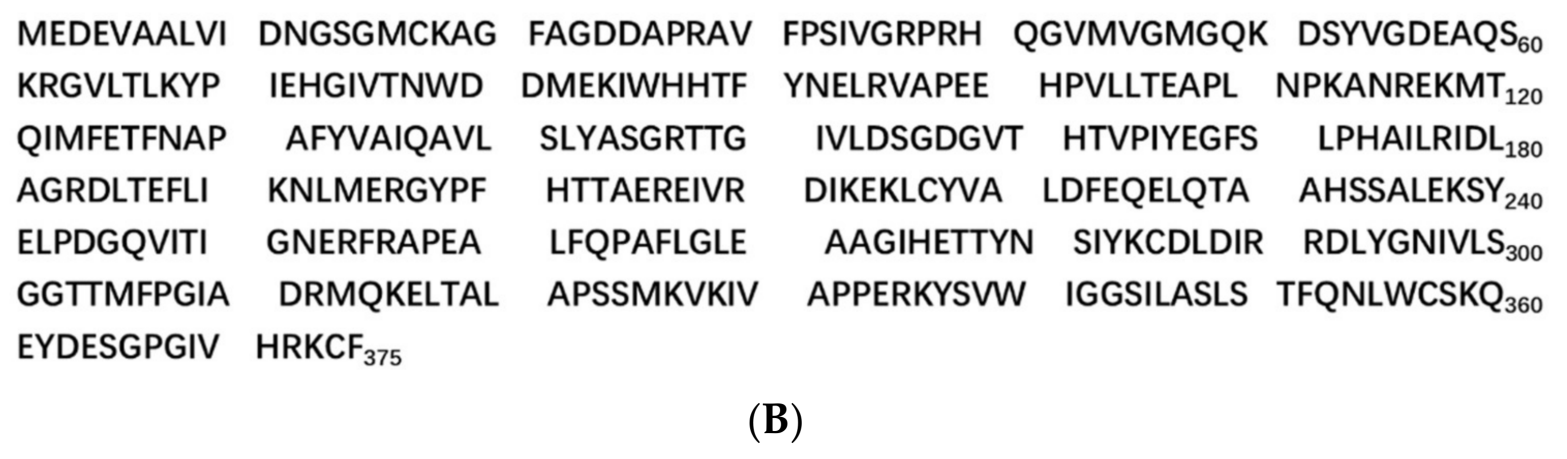
(**A**) The dominant polysaccharide structure of *FYGL* (Fudan-Yueyang-*G. lucidum*) characterized by chemical and multi-spectroscopic analysis [21]. Ara: arabinose, Gal: galactose, Glc: glucose, Rha: rhamnose. Thr: threonine, Ser: serine. Protein moieties are covalently bonded with carbohydrate moieties by Ser and Thr residues in –O– linkage. (**B**) The dominant sequence of the protein moieties of *FYGL* characterized by mass spectroscopy [22].

The mushroom *Ganoderma lucidum* has been used in traditional medicine for more than 2000 years in China to improve health, increase vitality, and extend life [14]. *Ganoderma lucidum* contains various bioactive compounds, such as polysaccharides, triterpenes, proteins, proteoglycans, sterols, fatty acids, and so on [15]. It has been proved to have anti-tumor, anti-diabetic, anti-inflammatory, and anti-oxidative functions [16,17,18,19]. In our previous study, we extracted a proteoglycan, named *FYGL* (Fudan-Yueyang-*G. lucidum*), from *Ganoderma lucidum* and found it to be a water-soluble, highly branched proteoglycan with a molecular weight of 2.6 × 10^5^ Da [20]. *FYGL* contains polysaccharides and covalently bonded protein by –O– linkage through Ser and Thr residues. The polysaccharide dominantly contains arabinose, galactose, glucose, rhamnose, etc., and the protein contains most natural amino acids, as shown in Figure 1, characterized by chemical and multi-spectroscopic analysis [21,22]. Previous work demonstrated that *FYGL* exhibited anti-hyperglycemic effects through the inhibition of both the activity and expression of protein tyrosine phosphatase 1B (PTP1B) [20,23], an insulin-resistant protein. However, the effect of *FYGL* on α-glucosidase and glycation is still unknown.

In this study, inhibition of α-glucosidase activity and non-enzymatic glycation by *FYGL* were evaluated both in vitro and in vivo. In vitro, the kinetic model of *FYGL* inhibiting α-glucosidase activity and the interaction between *FYGL* and α-glucosidase were investigated. Furthermore, the inhibitory effect of *FYGL* on non-enzymatic glycation was studied at three stages of the glycation process, identified by the formation of typical fructosamine, dicarbonyl compounds, and AGEs at the different stages, respectively, using the fructose-glycated model established in bovine serum albumin (BSA) [24]. Methylglyoxal (MGO) is one of the highly reactive dicarbonyl compounds generated at the middle stage of the glycation process [25] which was further used to establish a BSA glycated model, BSA-MGO model, to evaluate the effect of *FYGL* on the end stage of the glycation process. Simultaneously, the glycoxidation and fibrillation of proteins occurring in the glycation process and *FYGL*’s anti-glycation mechanism were investigated as well. In addition, to conform the results in vitro, the effect of *FYGL* in vivo on postprandial hyperglycemia was evaluated by oral sucrose tolerance test (OSTT) in *db/db* mice and that on diabetic vascular injury by histopathology and AGE accumulation in aortic tissues of diabetic rats. The present research would attempt to provide scientific evidence for *FYGL* as a novel anti-diabetic and anti-glycation agent used in diabetes management and treatment.

## 2. Results and Discussion

### 2.1. Anti-Postprandial Hyperglycemic Effect of FYGL In Vitro

#### 2.1.1. Inhibition Rate of α-Glucosidase by *FYGL*

**Figure 2:**
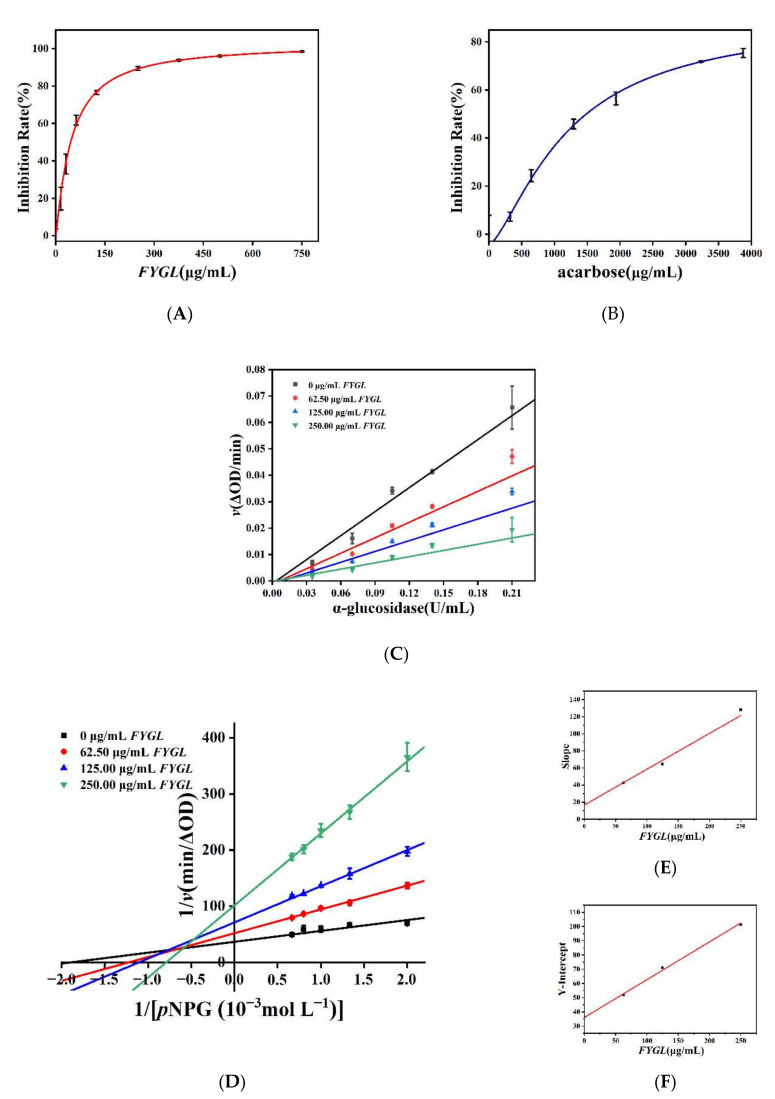
Inhibitory effect of *FYGL* (**A**) and acarbose (**B**) on α-glucosidase. (**C**) Dependence of enzymatic rate *v* vs. α-glucosidase concentration at different *FYGL* concentrations. (**D**) Lineweaver–Burk curves of 1/*v* vs. 1/[*p*NPG], effected by *FYGL*. Dependences of the slopes (**E**) and y-intercepts (**F**) in (**D**) vs. *FYGL* concentration. Each value represents the mean ± SD (*n* = 3).

The inhibitory capacity of *FYGL* and acarbose on α-glucosidase activity was studied using *p*-nitrophenyl-α-d-glucopyranoside (*p*NPG) as substrate. Figure 2A,B show the inhibition rate curves of *FYGL* and acarbose (positive control) at different concentrations. With the concentration of *FYGL* increased, the inhibition rate of α-glucosidase activity was increased. These results indicated that *FYGL* inhibited α-glucosidase activity in a dose-dependent manner. When the concentration reached 750 μg/mL, α-glucosidase activity was almost completely inhibited. Logistic fitting analysis for curves showed that the IC_50_ values of *FYGL* and acarbose were 45.45 μg/mL and 1485.32 μg/mL, respectively, implying that the inhibitory capacity of *FYGL* on α-glucosidase was greater than that of acarbose.

#### 2.1.2. Inhibitory Model of *FYGL* on α-Glucosidase Activity

To explore the inhibition model of *FYGL* on α-glucosidase, the kinetics were investigated. As shown in Figure 2C, the enzymatic reaction rate *v* increased as the enzyme concentration increased at different concentrations of *FYGL*, where the *v* value was the change in absorbance at 405 nm per min over 30 min. All of the fitted curves of the enzyme reaction rate at different *FYGL* concentrations passed through the origin, and the slopes of the lines decreased as the *FYGL* concentration increased, indicating that the inhibition of α-glucosidase was reversible and the intermolecular interaction between *FYGL* and α-glucosidase was non-covalent [26]. In addition, Lineweaver–Burk double reciprocal curves of the dependences of 1/*v* vs. 1/[*p*NPG] at different *FYGL* concentrations were plotted as Figure 2D, in which all of the lines intersected at a point of (−x, +y) in the orthogonal coordinates, where x, y > 0, indicating that *FYGL* caused a mixed-model inhibition, which suggested that *FYGL* could competitively bind to free α-glucosidase as well as noncompetitively to the enzyme–substrate complex. A similar inhibition effect was also found in sericin peptide [27], tannic acid [28], and some other natural products [29].

The mixed-model inhibition can be described by Lineweaver–Burk plots as Equation (1), and the slope and y-intercept plots can be described in Equations (2) and (3), respectively:

(1) $$\frac{1}{v}=\frac{K_{m}}{v_{\max}}\left(1+\frac{\left[I\right]}{K_{i}}\right)\frac{1}{\left[S\right]}+\frac{1}{v_{\max}}\left(1+\frac{\left[I\right]}{K_{\mathrm{is}}}\right)$$

(2) $$\mathrm{Slope}=\frac{K_{m}}{v_{\max}}+\frac{K_{m}}{v_{\max}}\frac{1}{K_{i}}\left[I\right]$$

(3) $$Y-\mathrm{intercept}=\frac{1}{v_{\max}}+\frac{1}{v_{\max}K_{\mathrm{is}}}\left[I\right]$$

where [I] and [S] denote the concentration of inhibitor and substrate, respectively; *v* is the enzyme reaction rate; and *K_m_*, *K_i_*, and *K_is_* represent the Michaelis–Menten constant, the dissociation constant between free α-glucosidase and inhibitor, and the dissociation constant between enzyme–substrate complex and inhibitor, respectively [27,30]. The kinetic parameters of *K_i_* and *K_is_* can be calculated by Equations (2) and (3).

Figure 2E,F show the linear dependences of the slopes and y-intercepts in Figure 2D vs. different concentrations of *FYGL*, respectively, which suggested that there was one site or one type of site in α-glucosidase interacting with *FYGL* [31]. Furthermore, the dissociation constant of *K_i_* and the dissociation constant of *K_is_* were calculated to be 39.60 μg/mL and 135.88 μg/mL, respectively, based on the slopes of Figure 2E,F, which suggested that *FYGL* was more likely to bind with free α-glucosidase and competitive inhibition played a dominant role. A similar inhibition model was also found in kaempferol [32] and tannic acid [28].

#### 2.1.3. Fluorescence Quenching of α-Glucosidase by *FYGL*

Fluorescence quenching of a fluorophore is generally caused by the reaction of the excited state, transformation of energy, formation of ground state complex, collision of molecules, and so on, leading to the decrease in fluorescence quantum yield. α-Glucosidase contains endogenous fluorophores, such as tryptophan, tyrosine, and phenylalanine residues, all of which are aromatic amino acids [33]. Herein, the fluorescence quenching of α-glucosidase effected by *FYGL* was investigated.

**Figure 3:**
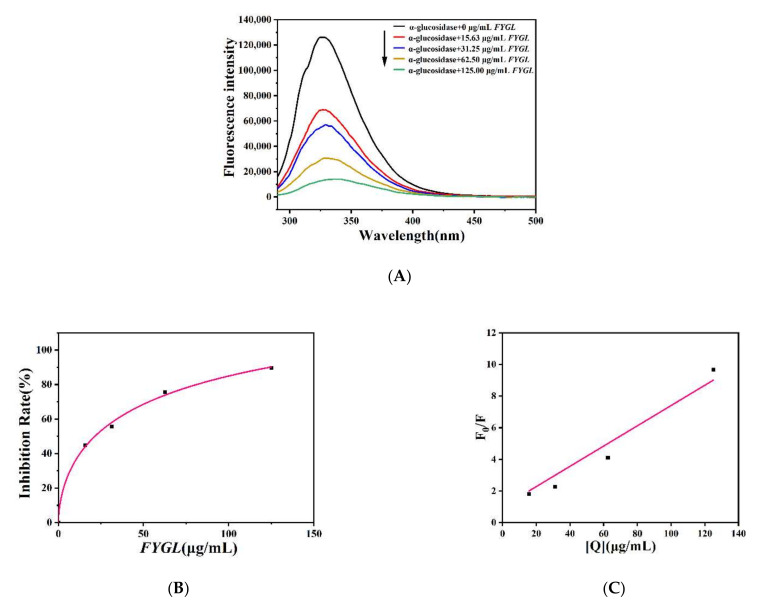

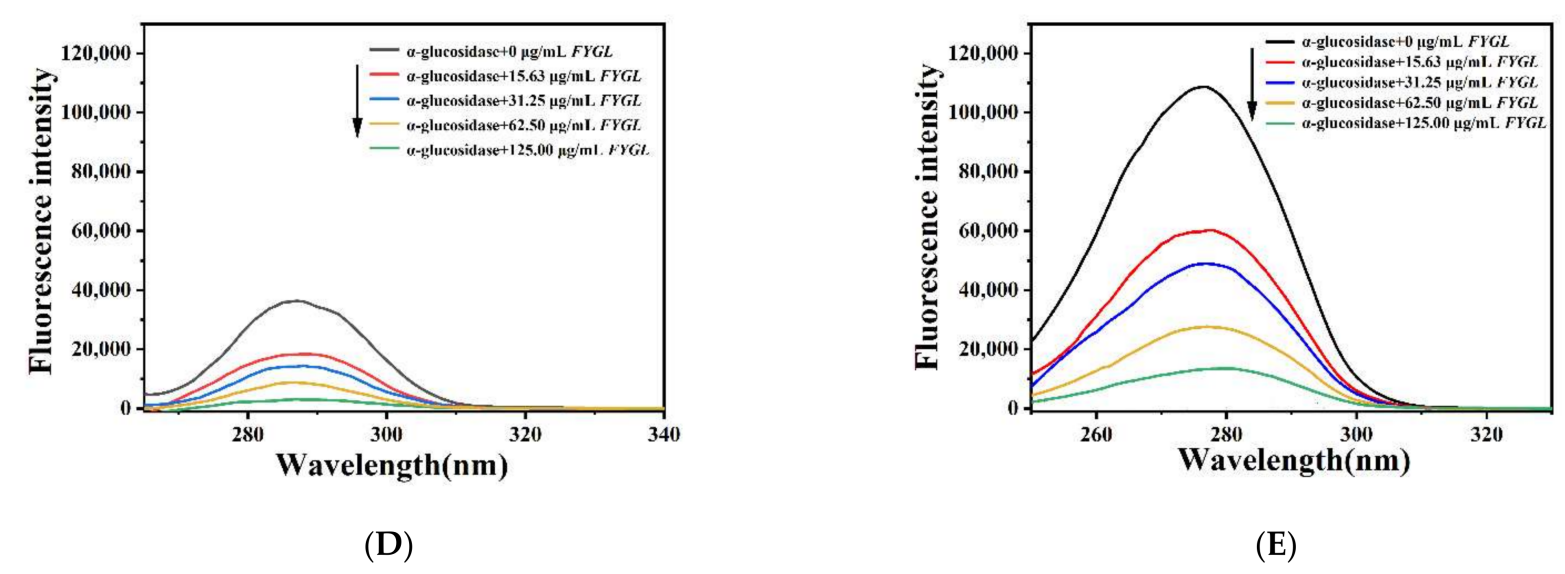
(**A**) Fluorescence spectra of α-glucosidase effected by *FYGL*. (**B**) The quench rate of fluorescence intensity of α-glucosidase effected by *FYGL* at maximum emission wavelength of 326 nm. (**C**) Stern–Volmer curve showing dependence of $\frac{F_{0}}{F}$ on concentration of quencher *FGYL*. (**D**,**E**) Synchronous fluorescence spectra of α-glucosidase with Δλ = 15 nm and 60 nm for tyrosine residue and tryptophan residue, respectively.

Figure 3A shows the fluorescence spectra of α-glucosidase effected by *FYGL* with excitation at 280 nm. As the concentration of *FYGL* increased, the fluorescence intensity of α-glucosidase gradually decreased. *FYGL* quenched the endogenous fluorescence of the enzyme in a concentration-dependent manner, indicating that *FYGL* interacted with α-glucosidase. Figure 3B shows the fluorescence quenching rate of α-glucosidase effected by *FYGL* at 326 nm, and the half-quenching concentration of *FYGL* on α-glucosidase fluorescence was calculated to be 21.46 μg/mL, which was consistent with its half-inhibition concentration (IC_50_) value detected in Section 2.1.1.

Fluorescence quenching generally has two types of model, static and dynamic. The former is induced by the formation of a complex without fluorescence between quencher and fluorophore, while the latter is induced by the collision between molecules, leading to the fluorescence decreasing. To investigate the fluorescence quenched model of α-glucosidase effected by *FYGL*, the fluorescence intensities at the maximum fluorescence emission wavelength were analyzed using the Stern–Volmer equation, Equation (4) [34]:

(4) $$\frac{F_{0}}{F}{=1+K}_{q}\tau_{0}\left[Q\right]$$

where *F*_0_ and *F* represent the fluorescence intensities in the absence and presence of *FYGL*, respectively; *K_q_* is the fluorescence quenching rate constant of α-glucosidase; *τ*_0_ is the average lifetime of α-glucosidase without the quencher of *FYGL*; and [Q] is the concentration of *FYGL*.

The fluorescence quenching rate constant *K_q_* can reflect the quenching model. If *K_q_* were much higher than 2.0 × 10^10^ L mol^−1^s^−1^, which is the maximum collision rate constant for all of the quenching agents that collide with biological macromolecules [35], the fluorescence quenching model would be thought to be a static one. Based on the Stern–Volmer equation (Equation (4)), where the average lifetime *τ*_0_ of a fluorophore is 10^−8^ s [36], *K_q_* was 1.7 × 10^18^ L mol^−1^s^−1^, calculated from the slope of the linear curve shown in Figure 3C, implying that the fluorescence quenching model of α-glucosidase effected by *FYGL* was dominantly a static one, which was consistent with the competitive-dominant inhibitory manner of *FYGL*, suggesting that a *FYGL*–α-glucosidase complex was formed.

#### 2.1.4. Synchronous Fluorescence Spectroscopy of α-Glucosidase Effected by *FYGL*

Synchronous fluorescence technology can make the overlapped spectra highly sensitive and resolved. It can detect the changes in the surrounding microenvironment of, for example, tyrosine and tryptophan residues in proteins [32]. Figure 3D,E show the effect of *FYGL* on the synchronous fluorescence spectra of α-glucosidase. The Δλ between the excitation and emission wavelengths was set to 15 and 60 nm to reveal the spectroscopic characteristics of tyrosine (Figure 3D) and tryptophan residues (Figure 3E), respectively [37]. The fluorescence intensity of tryptophan residues was stronger than that of tyrosine. *FYGL* quenched the fluorescence intensity of those two residues dose-dependently, and the quenching rate was faster for tryptophan than for tyrosine.

Overall, *FYGL* could bind with α-glucosidase to form *FYGL*–α-glucosidase complex and induce structural changes of α-glucosidase, resulting in the fluorescence quenching of amino acid residues. It has been reported that Asp215, 69, 352, Phe178, Val216, Glu411, Tyr72, 158, His112, Asn350, Gln353, and Arg442 are important amino acid residues in the catalytic center of α-glucosidase [37]. Some natural products, such as phloretin, can directly bind to Asp215, Arg442, and Gln353 residues in the active site of α-glucosidase and thereby inhibit its activity [37]. While some natural α-glucosidase inhibitors, such as tannic acid, do not bind to the active site of α-glucosidase, they bind to the nearby active sites and induce the conformation change of the enzyme [28]. Therefore, we speculate that *FYGL* could either bind to the active site of α-glucosidase or the nearby ones, inducing the structure and conformation changes of α-glucosidase and preventing the substrate entering the active site of the enzyme, thereby leading to the decrease of α-glucosidase activity.

### 2.2. Anti-Oxidative Capacity of FYGL

**Figure 4:**
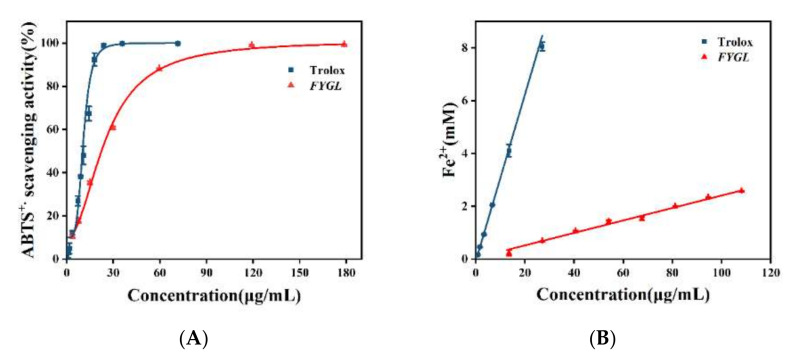
(**A**) ABTS^+•^ scavenging activity and (**B**) ferric-reducing power of *FYGL*. Trolox was used as the positive control. Each value represents the mean ± SD (*n* = 3).

The anti-oxidative capacity of *FYGL* was evaluated for its ability to scavenge ABTS^+•^ (2,2′-azino-bis (3-ethylbenzothiazoline-6-sulphonic acid cation radicals) and reduce Fe^3+^ ions. Trolox was used as the positive control. Figure 4A shows the ABTS^+•^ scavenging ability of *FYGL*. Logistic fitting analysis for curves showed that the IC_50_ values of *FYGL* and Trolox were 22.33 μg/mL and 10.18 μg/mL, respectively. Although the ability of *FYGL* scavenging ABTS^+•^ was slightly lower than that of Trolox, *FYGL* could scavenge most of ABTS^+•^ at higher concentration, implying that *FYGL* has strong anti-oxidative activity against free radicals. Figure 4B shows the power of *FYGL* reducing Fe^3+^ ions. Results showed that Trolox had much stronger reductive capacity for Fe^3+^ than *FYGL*, while the latter was much milder.

Overall, *FYGL* had strong radical-scavenging and mild ferric-reducing power; the electron donating moieties of *FYGL*, such as galactose and cystine, might contribute to this ability. In addition, the biological activities of natural products are highly related to the sites of glycosidic linkages and protein content in their chemical composition. For instance, a neutral heteropolysaccharide from *Lycium barbarum* containing mannose, galactose, and arabinose, linked by 1-3-, 1-2-, and 1-6-glycosidic linkages, was reported to have anti-oxidative activity [38]. A protein-bound heteropolysaccharide from tea was reported to have anti-oxidative activity with dependence on its protein content [39]. *FYGL* has the glycosidic linkages of 1-3-, 1-2-, and 1-6- and protein, which might be responsible for its anti-oxidative activity.

### 2.3. Anti-Glycation Effect of FYGL

#### 2.3.1. Inhibition of *FYGL* on Non-Enzymatic Protein Glycation in BSA-Fructose Model

##### Inhibition of *FYGL* on Three Stages of Glycation

The BSA-fructose model was used to evaluate the inhibitory effect of *FYGL* on non-enzymatic protein glycation in vitro. Aminoguanidine (AG) was used as the positive control. The process of non-enzymatic glycation of protein includes three stages: early stage, middle stage, and end stage. At the early stage, the amino groups in the proteins react with the carbonyl group in the reductive saccharides to form a Schiff base, which is unstable and prone to rearrangement to form relatively stable Amadori products; among them, fructosamine is the most representative one. At the middle stage, the Amadori products undergo dehydration, rearrangement, and other reactions to form highly reactive dicarbonyl compounds, such as the representatives of glyoxaldehydes and acetonaldehydes. At the end stage, the dicarbonyl compounds further react and crosslink with the free amino groups to form the complicated, stable, and irreversible complexes of advanced glycated end products (AGEs) [40]. For evaluating the capacity of anti-glycation at every stage of the non-enzymatic glycation process, the representative products can be detected by spectroscopy.

**Figure 5:**
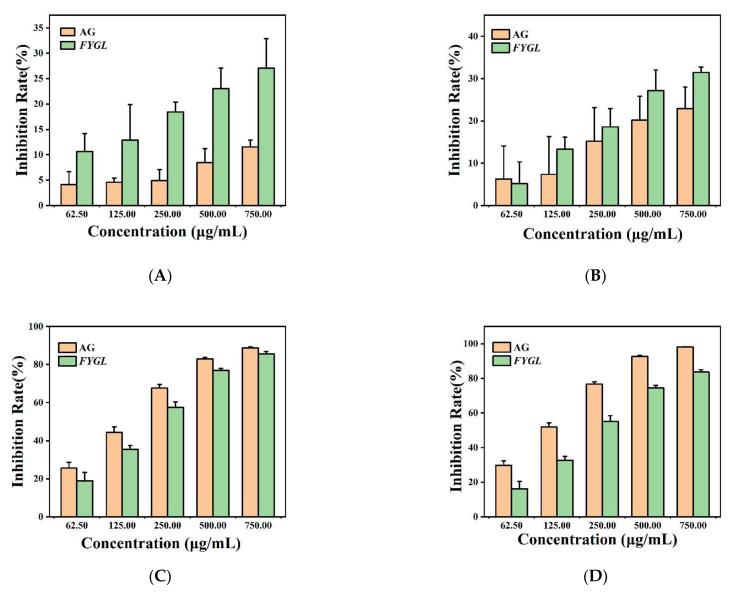

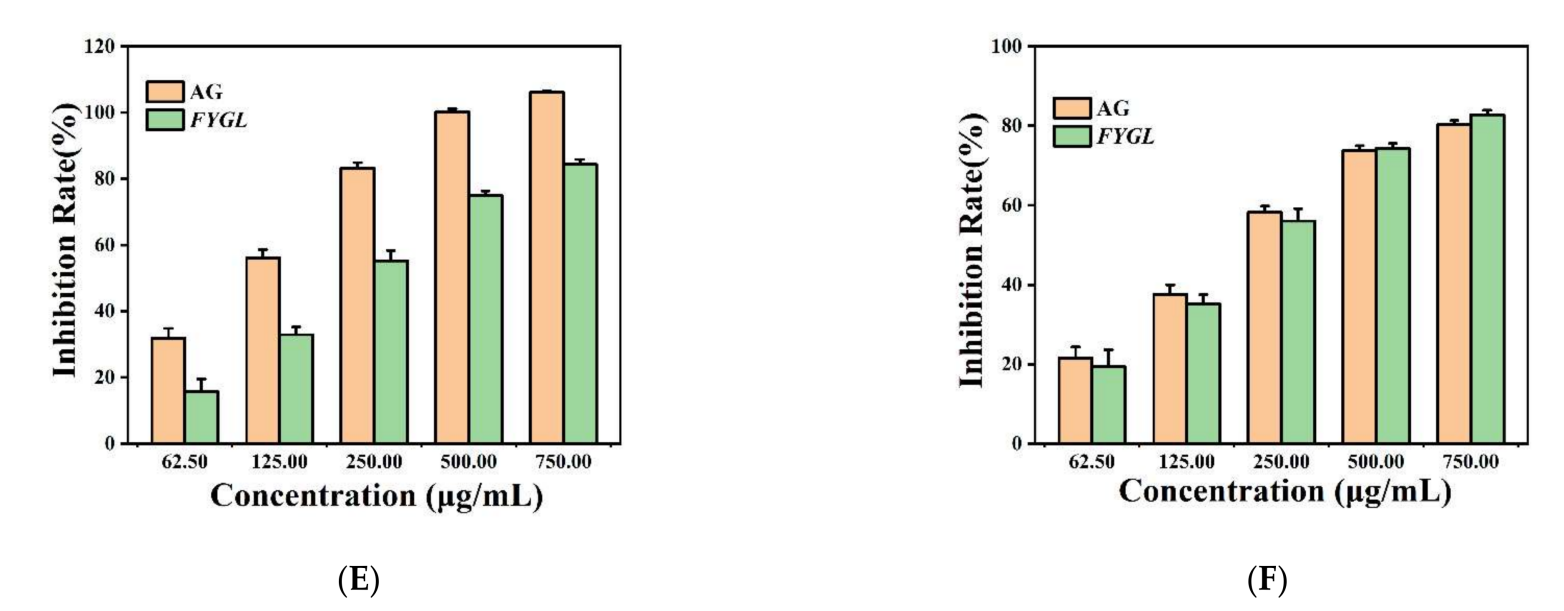
Effect of *FYGL* on the formation of glycation products in the bovine serum albumin (BSA)-fructose model. (**A**) Inhibition rate of fructosamine generated in the early stage in BSA-fructose sample incubated with *FYGL* or aminoguanidine (AG), measured by UV absorbance at 530 nm. (**B**) Inhibition rate of dicarbonyl compound generated in the middle stage, measured by UV absorbance at 290 nm. (**C**) Inhibition rate of advanced glycation end products (AGEs) generated in the end stage, measured by fluorescence emission at 440 nm with excitation at 350 nm. (**D**–**F**) Inhibition rates of formation of dityrosine, N’-formyl kynurenine, and kynurenine, respectively, measured by fluorescence at 415, 434, and 480 nm with excitation at 330, 325, and 365 nm, respectively. Each value represents the mean ± SD (*n* = 3).

Figure 5A shows the inhibition rate of fructosamine generated in the early stage in the BSA-fructose sample incubated with *FYGL* or AG, measured by UV absorbance at 530 nm using nitrotetrazolium blue chloride (NBT) assay [41]. The results showed that *FYGL* and AG suppressed the generation of fructosamine in a dose-dependent manner. The inhibition rate was increased from 12.91% to 27.08% as the concentration of *FYGL* increased from 125 to 750 μg/mL. The inhibition rate of AG (a positive control) was increased from 4.92% to 11.56% as the concentration of AG increased from 250 to 750 μg/mL. Compared to AG, *FYGL* had a higher inhibitory effect on the early stage of glycation. Figure 5B shows the inhibition rate of dicarbonyl compounds generated in the middle stage, measured by UV absorbance at 290 nm using Girard-T assay [42]. Results showed that *FYGL* and AG also decreased the generation of dicarbonyl compounds in a dose-dependent manner. The inhibition rate increased from 13.31% to 31.49% as *FYGL* concentration increased from 125 to 750 μg/mL and the inhibition rates of AG from 15.19% to 22.96% as AG concentration increased from 250 to 750 μg/mL. *FYGL* exerted an inhibitory effect stronger than AG in the middle stage of glycation. Figure 5C shows the inhibition rate of AGEs generated in the end stage measured by fluorescence emission at 440 nm with excitation at 350 nm [43]. *FYGL* showed excellently inhibitory capacity on the generation of AGEs. The inhibition rates increased from 35.41% to 85.68% as the concentration of *FYGL* increased from 125 to 750 μg/mL, and the generation of AGEs was almost completely suppressed at high concentration of *FYGL*.

As above, at *FYGL* concentration of 750 μg/mL, the inhibition rates of *FYGL* on glycation at the three stages were 27.08%, 31.49%, and 85.68%, respectively, indicating that the anti-glycation of *FYGL* was the most efficient at the end stage in the process of non-enzymatic glycation, during which the stable and irreversible complexes were formed.

##### Effect of *FYGL* on Protein Oxidation

Protein glycation is accelerated by oxidation. Dityrosine, N’-formyl kynurenine, and kynurenine are iconic products of protein glycoxidation; they result from the oxidation of tryptophan and tyrosine residues [44]. Figure 5D–F show the inhibition impact of *FYGL* and AG on the formation of these three markers of protein glycoxidation in the BSA-fructose sample, detected by their intrinsic fluorescence at 415, 434, and 480 nm with excitation at 330, 325, and 365 nm, respectively. A similar dose-dependent inhibition effect on three markers was observed, even though the inhibition rate of *FYGL* was slightly lower than that of AG. The results indicated that *FYGL* prevented the oxidation of tryptophan and tyrosine residues in BSA incubated with fructose. The outstanding anti-glycoxidation ability of *FYGL* also enhanced its anti-glycation capacity.

##### Inhibition of *FYGL* on Crosslinking and Aggregation of Amyloid Fibrillation

During glycation, amino acid residues in proteins, especially lysine and arginine residues, are modified by sugars, which induce the crosslinking and aggregation of the glycated proteins to ultimately form high-molecular-mass aggregates and amyloid-like fibrils in the later stages of glycation [45].

**Figure 6:**
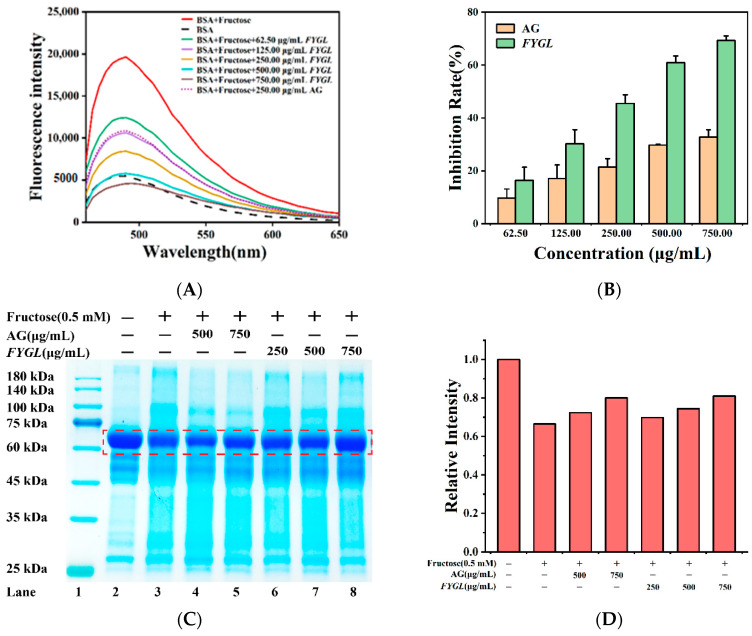
Effect of *FYGL* on crosslinking and aggregation in the BSA-fructose model. (**A**) The fluorescence spectra and (**B**) the inhibition rate on amyloid fibrillation formed in BSA-fructose sample incubated with *FYGL* or AG, based on the thioflavin T (ThT)-labeled fluorescence assay. Each value represents the mean ± SD (*n* = 3). (**C**) SDS-PAGE bands of BSA and glycated BSA in the presence or absence of *FYGL* or AG under denaturing conditions. Lanes from left to right represent the protein markers (lane 1), pure BSA (lane 2), glycated BSA (lane 3), glycated BSA with AG (lane 4, 5), and glycated BSA with *FYGL* (lane 6–8). (**D**) The relative image intensity of pure BSA band in red rectangles in (**C**).

Formation of BSA β-amyloid fibrillation was determined herein by thioflavin T (ThT)-labeled fluorescence assay. The greater the amyloid fibrillation, the higher the fluorescence intensity of ThT-labeled proteins. Figure 6A,B show the fluorescence spectra and the inhibition rate of amyloid fibrillation formed in the BSA-fructose sample incubated with *FYGL* or AG, respectively. Results showed that the fluorescence intensity was much higher in fructose-glycated BSA than that of pure BSA (Figure 6A), indicating the formation of aggregates of amyloid fibrils during glycation. Interestingly, the inhibitory rate of *FYGL* on aggregation increased in a dose-dependent manner, up to 69.28% at 750 μg/mL, much higher than the 32.73% of AG at the same concentration.

The molecular mass of aggregates formed during glycation were evaluated by SDS-PAGE. Figure 6C shows the SDS-PAGE gel of BSA and glycated BSA in the presence or absence of *FYGL* or AG under denaturing conditions. Results showed that pure BSA exhibited a clear single band at about 66 kDa; however, when BSA was glycated by fructose, a high-molecular-weight band was found. As shown in Figure 6D, the image intensity of the BSA band decreased after glycation, but *FYGL* markedly suppressed the decrease. Combined with the ThT-labeled fluorescence analysis, we concluded that *FYGL* inhibited protein crosslinking and aggregation during glycation.

#### 2.3.2. Preventative Effect of *FYGL* from BSA Glycation Induced by MGO

**Figure 7:**
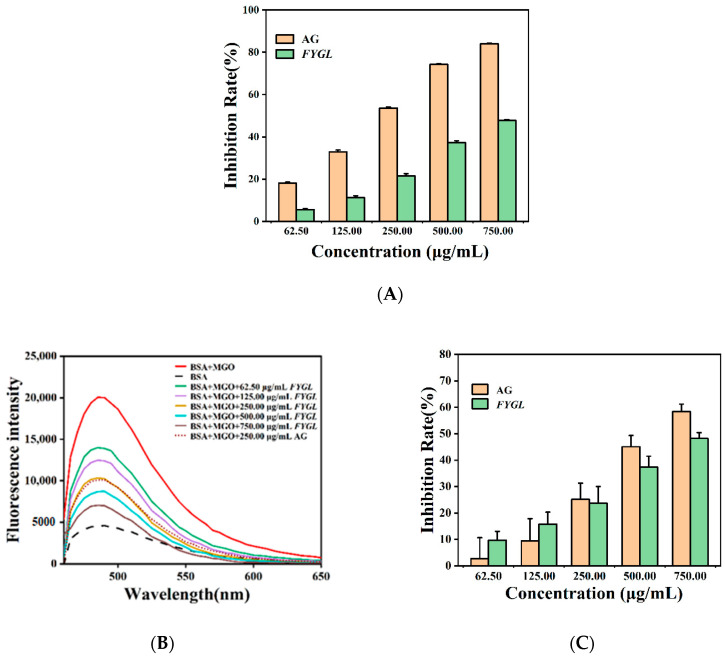
Effect of *FYGL* on glycation in BSA-methylglyoxal (MGO) model. (**A**) Inhibition rate of AGEs generated in the sample incubated with *FYGL* or AG, measured by intrinsic fluorescence. (**B**) Fluorescence spectra and (**C**) inhibition rate of amyloid fibrillation in the sample incubated with *FYGL* or AG, based on ThT-labeled fluorescence assay. Each value represents the mean ± SD (*n* = 3).

Methylglyoxal (MGO) is one of the most reactive dicarbonyl intermediates generated in the middle stage of protein glycation. MGO covalently binds to functional groups of proteins and induces free radicals, leading to the formation of AGEs and crosslinked aggregates at the end stage of protein glycation. To identify the influence of *FYGL* on the end stage of protein glycation in the BSA-MGO model, the inhibition on AGEs was measured by intrinsic fluorescence and that on β-amyloid fibrillation by ThT-labeled fluorescence. As shown in Figure 7, *FYGL* also strongly inhibited the generation of AGEs and alleviated β-amyloid fibrillation in the MGO-induced glycation model.

It has been reported that free radicals and oxidation could accelerate AGE formation [46]. In this study, *FYGL* possessed abilities of scavenging free radicals and reduction. Therefore, *FYGL* strongly inhibited the generation of AGEs found in both BSA-fructose and BSA-MGO models, potentially leading to the alleviation of AGEs-related diabetic complications. Similarly, some natural products, such as Chinese bayberry (*Myrica rubra*) phenolics [47] and *Gynura procumbens* polysaccharides [48], have anti-glycation capacity due to their anti-oxidative activities.

#### 2.3.3. Fluorescence Quenching of BSA by *FYGL*

**Figure 8:**
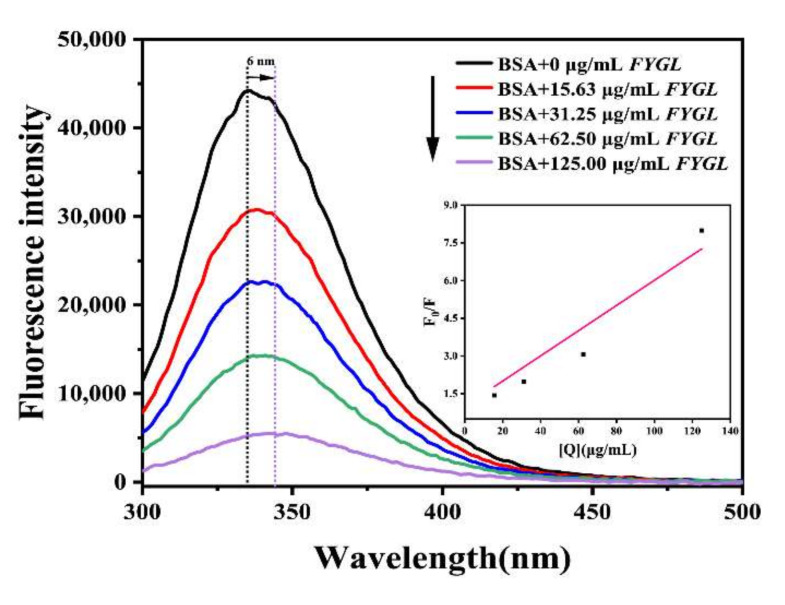
Fluorescence spectra of BSA effected by *FYGL*. The inset plot is the Stern–Volmer curve showing dependence of $\frac{F_{0}}{F}$ on concentration of quencher *FGYL*.

To reveal the interaction between *FYGL* and BSA in the anti-glycation function in the BSA-glycated model, the intrinsic fluorescence quenching of BSA was recorded. Figure 8 shows the fluorescence spectra of BSA effected by *FYGL* with excitation at 280 nm. As the concentration of *FYGL* increased, the fluorescence intensity of BSA gradually decreased and the maximum fluorescence emission wavelength was red-shifted by 6 nm, indicating that *FYGL* interacted with BSA to lead to an increase in the polarity and hydrophilicity in the micro-environment of tryptophan residues [49]. Similarly, based on the Stern–Volmer equation (Equation (4)), the fluorescence quenching rate constant *K_q_* was 1.3 × 10^18^ L mol^−1^s^−1^ from the inset graph in Figure 8, implying that the fluorescence quenching model was also a static one and a stable complex of *FYGL*–BSA was formed. Recently, some studies have reported that formation of non-covalent complexes with proteins can restrain the glycation [47,49]. Moreover, the hydrogen bonds played an important role to stabilize the non-covalent complex structure [50]. *FYGL* is a hydroxy-enriched proteoglycan and could possibly form a stable *FYGL*–BSA complex to hinder fructose or MGO from binding to active residues, such as lysine and arginine residues in BSA [47], thereby inhibiting BSA glycation.

### 2.4. Effect of FYGL on OSTT In Vivo

**Figure 9:**
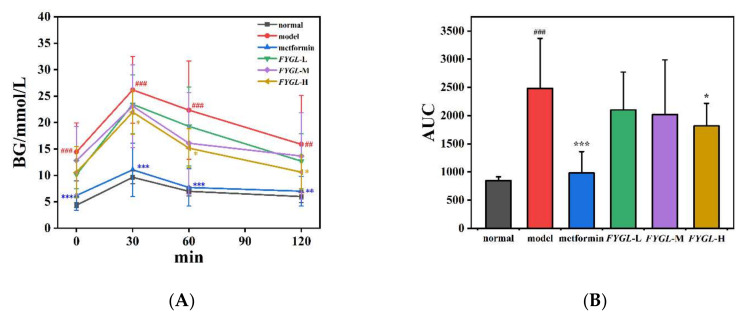
Postprandial blood glucose (PBG) affected by oral administration of *FYGL* in sucrose-loaded normal mice and diabetic mice. (**A**) PBG levels measured after oral administration of sucrose over 120 min. (**B**) The value of the area under the curve (AUC) of each group calculated from the curve areas in (**A**). The values represent the mean ± SD (*n* = 12; ^###^ *p* < 0.001 vs. normal, ^##^ *p* < 0.01 vs. normal, * *p* < 0.05 vs. model, ** *p* < 0.01 vs. model, *** *p* < 0.001 vs. model).

We have previously demonstrated that *FYGL* significantly alleviated the fasting blood glucose (FBG) level, a major indicator of hyperglycemia, in *db/db* mice [51]. In this study, we focused on the postprandial blood glucose (PBG) level, the other indicator of hyperglycemia, influenced by *FYGL* through oral sucrose tolerance test (OSTT). The normal and diabetic mice were randomly assigned into six groups, including normal group; model group; metformin group treated with 225 mg/kg metformin; *FYGL*-L group treated with 225 mg/kg *FYGL*; *FYGL*-M group treated with 450 mg/kg *FYGL* and *FYGL*-H group treated with 900 mg/kg *FYGL*. Figure 9A,B show the anti-postprandial hyperglycemic effect of *FYGL* on sucrose-loaded normal and *db/db* mice over 120 min with metformin as a positive reference. The results show that the peak values of blood glucose in each group appeared at approximately 30 min after oral administration of sucrose and then dropped. The blood glucose concentrations of *db/db* mice (model group) remained higher than that of normal mice. For *FYGL* groups, the blood glucose values were lower than that of the model group after 30 min, and that of the high-dose *FYGL*-H group was significantly lower, indicating that *FYGL* could efficiently reduce PBG. The AUC results in Figure 9B were consistent with those in Figure 9A. The results suggested that *FYGL* effectively alleviated sucrose-mediated PBG in *db/db* mice, possibly because of the inhibition of α-glucosidase activity.

### 2.5. Effect of FYGL Treatment on Aortic Histopathology and AGE Accumulation In Vivo

**Figure 10:**
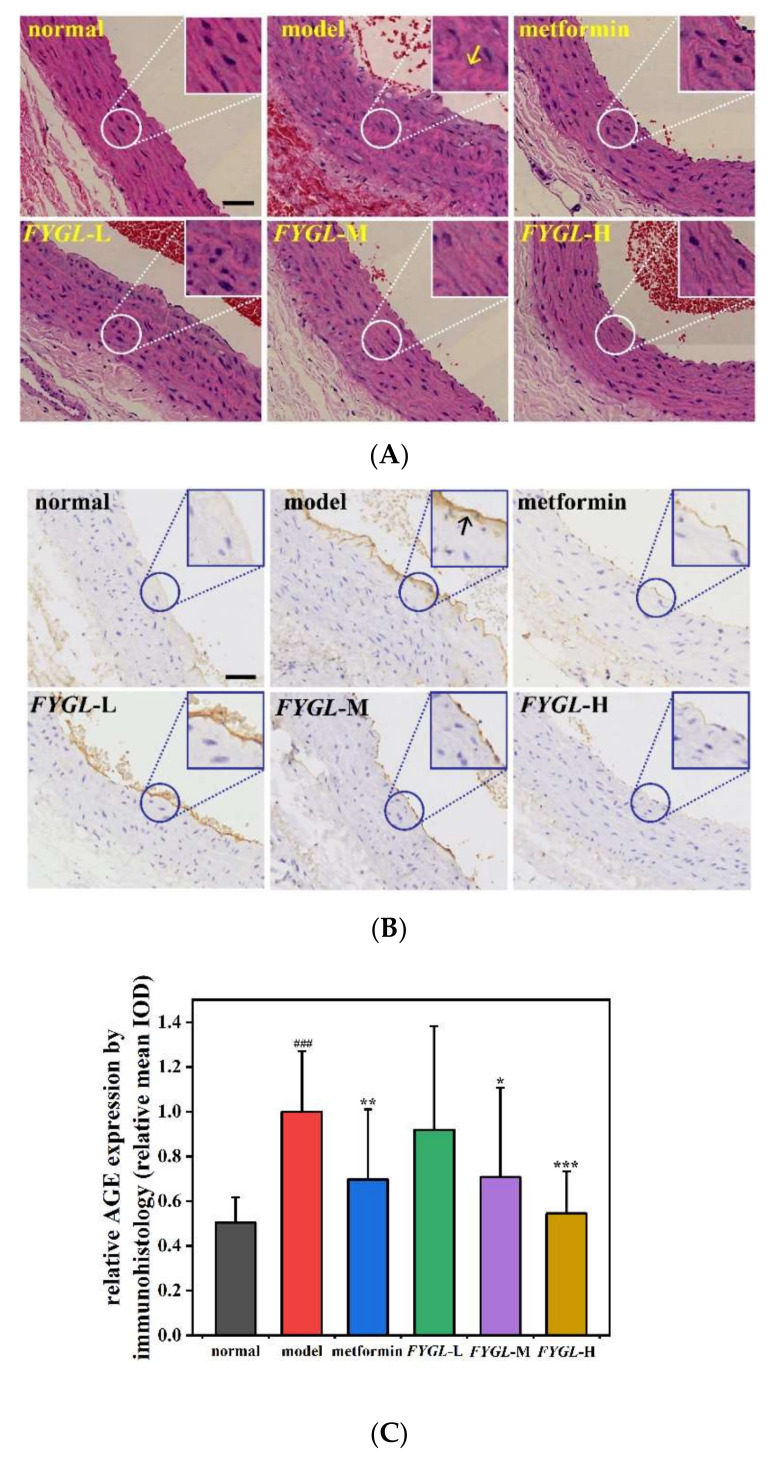
Histopathology and AGE accumulation in aorta tissues in vivo. (**A**) Representative images of H&E-stained aorta tissues, magnification 100×. The yellow arrow shows the aorta layers (in red) severely disordered and the nuclei (in dark blue) unevenly distributed in model group. (**B**) The immunohistochemical analysis of AGEs in the aorta tissues, magnification 100×. The black arrow shows the heave AGE accumulation (in brown) in model group. The scale bar is 50 μm. The typical images in (**A**,**B**) were enlarged by four times for clear observation. (**C**) Quantitative analysis of AGE accumulation in the aorta by Image-Pro Plus 6.0 software. The values represent the mean ± SD (*n* = 6; ^###^ *p* < 0.001 vs. normal, * *p* < 0.05 vs. model, ** *p* < 0.01 vs. model, *** *p* < 0.001 vs. model).

*FYGL* can alleviate the level of biochemical indexes, such as FBG, triglycerides, and total cholesterol, in STZ-induced diabetic SD rats [23]; these indexes are related to vascular injury and AGE accumulation. In this study, to evaluate the effect of *FYGL* treatment on diabetic vascular injury, H&E staining was applied to observe the morphological changes of abdominal aortic tissues in normal and diabetic SD rats (Figure 10A). The grouping information was similar as Section 2.4, but dosages are for SD rats. In the normal group, both the intimal and medial layers of the H&E-stained aorta showed orderly arrangement (in red), and the nuclei showed uniform distribution (in dark blue) without deformation and lesion. In the model group, both intimal and medial layers were disordered and thickened, while after oral treatment of *FYGL* and metformin (a positive drug), the aorta layers were orderly arranged with less deformation, and the nuclei were evenly distributed, indicating that *FYGL* could alleviate the vascular injury.

In addition, because AGE accumulation could result in the injury of aortic tissues, the expression of AGEs was measured in vivo by immunohistochemical analysis shown in Figure 10B,C. The results showed that the AGE expression (in brown) was greatly enhanced in the model group compared to the normal group, while it was significantly alleviated in a dose-dependent manner after *FYGL* treatment, possibly due to *FYGL* preventing protein crosslinking and fibrillation, thereby preventing injury of the vasculature. Furthermore, AGEs can activate the AGE-RAGE pathway, resulting in oxidative stress and inflammation [52], while *FYGL* could potentially inhibit activation of the AGE-RAGE pathway and alleviate related diabetic complications.

## 3. Materials and Methods

### 3.1. Materials

Fruiting bodies of *Ganoderma lucidum* grown in north-eastern China were purchased from Leiyunshang Pharmaceutical Co. Ltd. (Shanghai, China). *FYGL* was obtained from *Ganoderma lucidum* as previously described [20], and Figure S1 shows its NMR spectrum. Yeast α-glucosidase (EC 3.2.1.20), purchased from Sigma-Aldrich Co. (St. Louis, MO, USA), was prepared in sodium phosphate buffer (0.1 M, pH 6.8). Metformin, acarbose, aminoguanidine (AG), *p*-nitrophenyl-α-D-glucopyranoside (*p*NPG), nitrotetrazolium blue chloride (NBT), Girard-T reagent, thioflavin T (ThT), and methylglyoxal (MGO) were purchased from Aladdin Chemical Co. (Shanghai, China). Fructose, sucrose, and trichloroacetic acid (TCA) were purchased from Sinopharm Chemical Reagent Co., Ltd. (Shanghai, China). The antibody of AGEs was purchased from Abcam (Cambridge, MA, USA). Bovine serum albumin (BSA) was obtained from Yeasen biotechnology Co., Ltd. (Wuhan, China). All other chemicals and solvents were of analytical quality, and fresh ultrapure water was used in all of the experiments.

### 3.2. Anti-Postprandial Hyperglycemia Assay In Vitro

#### 3.2.1. Inhibition of α-Glucosidase Activity

The α-glucosidase inhibition potency of *FYGL* was evaluated using the method reported by Han [37], with minor modifications. Briefly, the α-glucosidase enzyme activity was measured by adding 50 μL of 0.07 unit/mL α-glucosidase from S. cerevisiae and 50 μL of 1.0 mM *p*NPG in 0.1 M phosphate buffer solution (pH 6.8), with or without *FYGL*, and acarbose as a positive control. The UV absorbance at 405 nm of the mixed solution was recorded every 60 s at 37 °C with a microplate reader. The enzyme activity was presented as the change in the absorbance per minute, which was the slope of the obtained curve. The inhibitory effect of *FYGL* or acarbose was defined as Equation (5):

(5) $$\mathrm{Inhibitory}\;\mathrm{activity}\;\left(\%\right)=\frac{R_{\mathrm{blank}}-{\;R}_{\mathrm{inhibitor}}}{R_{\mathrm{blank}}}\;\times \;100$$

where R_inhibitor_ is the slope of the reaction kinetics curve obtained by the reaction of inhibitor of *FYGL* or acarbose with substrate, and R_blank_ is the slope without inhibitor. To evaluate the inhibitory potency of the inhibitors, the concentrations of *FYGL* and acarbose leading to the loss of 50% α-glucosidase activity (IC_50_) was calculated based on the logistic analysis of the curve of inhibition rate vs. concentration [53].

#### 3.2.2. Inhibition Model on α-Glucosidase

To investigate the inhibition model of *FYGL* on α-glucosidase, different concentrations of *FYGL* were individually added to the mixtures of 50 μL of α-glucosidase within concentrations of 0.035–0.210 unit/mL and 50 μL of *p*NPG within concentrations of 0.5–1.5 mM. The absorbance of the reaction mixture was recorded every 60 sec at 405 nm. The dependence of enzymatic rate *v* vs. α-glucosidase concentration at different *FYGL* concentrations was used to determine the inhibition model. The *v* value was the change in absorbance at 405 nm per min. The inhibition kinetics parameters were analyzed using double-reciprocal plots (Lineweaver–Burk plots) of the inhibition rate *v* vs. substrate concentration, and the plots of the slopes and y-intercepts vs. *FYGL* concentration.

#### 3.2.3. Intrinsic Fluorescence Quenching Assay of α-Glucosidase

To further investigate the interaction between *FYGL* and α-glucosidase, the intrinsic fluorescence of α-glucosidase was recorded after incubation with *FYGL*. α-Glucosidase (0.05 mg/mL) was pretreated with various concentrations of *FYGL* (15.63–125.00 μg/mL) for 10 min at room temperature. Then, the intrinsic fluorescence spectra of α-glucosidase were recorded by FLS1000 (Edinburgh Instruments, Livingston, UK) at emission wavelengths in the range of 300–500 nm with an excitation wavelength at 280 nm at room temperature. The excitation and emission bandwidths were both set to 1.0 nm. The fluorescence intensities of α-glucosidase affected by *FYGL* at the maximum fluorescence emission wavelength were analyzed using the Stern–Volmer equation.

The synchronous fluorescence spectroscopy is a novel technology which can sensitively identify the fluorescence spectrum of different amino residues in protein by changing the interval wavelength (Δλ) between excitation and emission [32]. Herein, the synchronous fluorescence spectra were recorded at Δλ of 15 and 60 nm to characterize the fluorescence of tyrosine and tryptophan, respectively. The excitation and emission slit widths were both set to 1.0 nm.

### 3.3. Anti-Oxidative Assay In Vitro

To investigate the anti-oxidative capacity of *FYGL*, the scavenging activity of a radical cation of ABTS^+•^ and the reduction power were investigated.

The ability of *FYGL* to scavenge ABTS^+•^ was assayed by a total antioxidant capacity assay kit of ABTS (Beyotime Biotechnology Development Co., Ltd., Shanghai, China). Briefly, 200 μL of fresh ABTS radical cation solution was mixed with 10 μL *FYGL* at room temperature for 6 min, and then the absorbance was recorded at 734 nm. Trolox was used as a positive control. The ABTS^+•^ scavenging activity was calculated using Equation (6):

(6) $$\mathrm{Scavenging}\;\mathrm{activity}\;\left(\%\right)=\frac{{\mathrm{OD}}_{\mathrm{blank}}-{\;\mathrm{OD}}_{\mathrm{test}}}{{\mathrm{OD}}_{\mathrm{blank}}}\;\times \;100$$

where OD_test_ represents the optical density (OD) of the mixture with *FYGL* or Trolox and OD_blank_ denotes that of blank solution without *FYGL* or Trolox.

The reduction power was tested using a total antioxidant capacity assay kit with Fe^3+^ (Beyotime Biotechnology Development Co. Ltd., Shanghai, China). Briefly, 180 μL of ferric-tripyridyltriazine solution was mixed with 5 μL *FYGL* at 37 °C for 5 min, and then the absorbance was recorded at 593 nm. Trolox was used as the positive control. The reduction power of the samples was expressed as mmol Fe^2+^. The higher the content of Fe^2+^, the stronger the reduction capacity of *FYGL*.

### 3.4. Anti-Glycation Assay In Vitro

#### 3.4.1. BSA-Fructose Model of Non-Enzymatic Protein Glycation

The BSA-fructose model was established based on the work reported by Wang [54] with minor modification to evaluate the inhibitory effect of *FYGL* on the glycation process. AG (aminoguanidine) was used as the positive control. BSA (20.0 mg/mL) and different concentrations of *FYGL* or AG were dissolved in potassium phosphate buffer (pH 7.4, 0.1 M) containing 0.02% sodium azide and pre-incubated for 30 min at room temperature, and then fructose (0.5 M) was added and incubated at 50 °C for 24 h. After incubation was terminated, the sample was stored at −20 °C for further use.

##### NBT Assay for Fructosamine

The content of fructosamine in the BSA-fructose sample was analyzed according to the NBT (nitrotetrazolium blue chloride) method reported by Johnson [41], during which NBT could be reduced by fructosamine to form a colored formazan with strong UV absorption at 530 nm. BSA-fructose solution and 0.3 mM NBT solution were incubated at room temperature for 15 min and then transferred to 96-well plates; UV absorbance of the mixture solution was recorded at 530 nm to quantify the concentration of fructosamine. The inhibitory effect of *FYGL* was determined using Equation (7):

(7) $$\mathrm{Inhibitory}\;\mathrm{rate}\;\left(\%\right)=\frac{{\mathrm{OD}}_{\mathrm{blank}}-{\;\mathrm{OD}}_{\mathrm{test}}}{{\mathrm{OD}}_{\mathrm{blank}}}\;\times \;100$$

where OD_test_ represents the OD of the sample with *FYGL* or AG and OD_blank_ denotes that of blank solution without *FYGL* or AG.

##### Girard-T Assay for Dicarbonyl Compound

The content of dicarbonyl compounds in the BSA-fructose sample was analyzed according to the Girard-T assay reported by Wells-Knecht [42], in which Girard-T reagent can react with dicarbonyl compound to form an addition product with strong absorption at 290 nm. In brief, BSA-fructose solution, 0.5 M Girard-T reagent, and 0.5 M sodium formate solution were mixed at room temperature for 1 h. The UV absorbance of the mixture solution was recorded at 290 nm to determine the dicarbonyl compound concentration. The inhibitory effect of *FYGL* was calculated using Equation (7).

##### Fluorescence Assay for Generated AGEs and Oxidation Products

The contents of AGEs produced in the BSA-fructose samples were analyzed by fluorescence at 440 nm with excitation at 350 nm [43]. In addition, the representative protein oxidation products of dityrosine, N-formyl kynurenine, and kynurenine were also measured by fluorescence at 415, 434, and 480 nm with excitation at 330, 325, and 365 nm, respectively [55]. The related inhibition rate was calculated using Equation (8):

(8) $$\mathrm{Inhibitory}\;\mathrm{rate}\;\left(\%\right)=\frac{F_{0}-\;F}{F_{0}}\;\times \;100$$

where *F*_0_ represents the fluorescence intensity without *FYGL* or AG and *F* represents that with *FYGL* or AG.

##### ThT-Based Fluorescence Assay for β-Amyloid Protein Aggregate

Thioflavin T (ThT) fluorescence would increase if ThT interacted with β-amyloid protein aggregate; therefore, it was used to identify the amount of β-amyloid protein aggregates [56]. Glycated protein was incubated with 20 μg/mL ThT solution at 25 °C for 60 min. Then, the fluorescence spectra of the mixed solution were recorded at an emission wavelength range of 455–600 nm with excitation wavelength of 435 nm, and the intensity was recorded at 485 nm for amount of β-amyloid protein aggregates.

##### SDS-PAGE Assay for Protein Aggregation

Protein molecular weight was analyzed via the SDS-PAGE method according to Eze [57]. The glycated protein samples were dissolved and denatured with loading buffer at 100 °C for 5 min after being centrifugated at 12,000 rpm to obtain protein precipitate by adding TCA (trichloroacetic acid). Then, 10 μg denatured protein was separated by SDS-PAGE with 10% gel. The SDS-PAGE gel was dyed with Coomassie Blue super-fast staining solution (Beyotime Biotechnology Development Co., Ltd., Shanghai, China) for 30 min and then decolorized overnight. The protein content of each band on the stained gel was semi-quantified using Image J software.

#### 3.4.2. BSA-MGO Model of Non-Enzymatic Protein Glycation

To further assess the effect of *FYGL* on the end stage of glycation process, the BSA-MGO model was established according to the literature with minor modifications [58]. The experiment procedure was similar to that of the BSA-fructose model, except that, afterwards, MGO (5.0 mg/mL) was added. The BSA-MGO sample was incubated at 37 °C for 72 h in the dark. Then, the contents of AGEs and β-amyloid protein aggregate produced in the BSA-MGO samples were analyzed using the same method mentioned in Section 3.4.1.

#### 3.4.3. Intrinsic Fluorescence Quenching Assay of BSA

To reveal the interaction between *FYGL* and BSA in the anti-glycation function in BSA-glycated models, the intrinsic fluorescence of BSA was recorded after incubation with *FYGL*, referring to the protocols reported by Li [59]. BSA (0.1 mg/mL) was pretreated with various concentrations of *FYGL* (15.63–125.00 μg/mL) at 37 °C for 2 h, and then the intrinsic fluorescence spectra were detected at emission wavelengths in the range of 300–500 nm, with excitation wavelength at 280 nm at room temperature. The excitation and emission bandwidths were both set to 3.0 nm. The fluorescence intensities at the maximum fluorescence emission wavelength were analyzed using the same Stern–Volmer equation (Equation (2)).

### 3.5. Animal Trial

#### 3.5.1. OSTT in Mice

Male, four-week-old, BKS normal mice and BKS-*db* (*db/db*) diabetic mice were purchased from Gempharmatech Co., Ltd., Nanjing, China. The animals were housed in the SPF Animal Experimental Center of Efficacy Evaluation, School of Pharmacy, Fudan University. Mice were free to access water and food in the house maintained at a temperature of 20–26 °C and humidity of 40–70% with 12 h light/dark cycle. All trials and the care of animals were conducted under the guidance of the Experimental Animal Ethics Committee of Fudan University. After 1 week of adaptation, blood was collected from the tail vein of each mouse, and the fasting blood glucose level was measured. Only animals with blood glucose concentration higher than 11.1 mM were used as the diabetic model for the trials.

Normal and diabetic mice were randomly assigned into 6 groups, with 12 mice per group, and orally administrated the drugs. Six groups included: normal group (normal BKS mice treated with saline); model group (diabetic mice treated with saline); metformin group (mice treated with metformin, 225 mg/kg); *FYGL*-L group (diabetic mice treated with a low dose of *FYGL*, 225 mg/kg); *FYGL*-M group (diabetic mice treated with a medium dose of *FYGL*, 450 mg/kg); and *FYGL*-H group (diabetic mice treated with a high dose of *FYGL*, 900 mg/kg). The fasting blood glucose level was measured weekly. To identify the effect of *FYGL* on postprandial blood glucose (PBG) in vivo, an oral sucrose tolerance test (OSTT) was used to reflect PBG after the mice were treated with drugs for 8 weeks. The normal and diabetic mice were orally administered 2.5 g/kg sucrose after 2 h of drugs or saline taken, and blood samples were collected from the tail vein at 0, 30, 60, and 120 min. PBG concentration was determined using a Contour TS blood glucose meter (Bayer, Berlin, Germany). The area under the curve (AUC) of blood glucose value vs. time was calculated to quantitatively identify the PBG.

#### 3.5.2. Histopathology and Immunohistology of Aortic Tissues in Diabetic Rats

Because the abdominal aortas from *db/db* mice were very thin, it is difficult to observe their morphology and investigate the accumulation of AGEs affected by the drugs. The diabetic rats were used for this purpose.

Male, four-week-old, SD rats were purchased from Shrek Laboratory Animal Co., Ltd., Shanghai China. The animals were housed in the same surroundings as Section 3.5.1. After 1 week of adaptation, all rats were randomly assigned either to a diabetic group or to a healthy normal group. The normal group rats were fed standard diet, while the rats in the diabetic group were fed a high-fat diet for 1 month. The diabetic group was intraperitoneally injected with a single dose of streptozotocin (STZ, 40 mg/kg of bodyweight; Sigma-Aldrich, St. Louis, MO, USA) to establish a diabetes mellitus (DM) model with blood glucose levels > 16.7 mmol/L. The diabetic group was further divided into five groups, including model group; metformin group treated with 200 mg/kg metformin; *FYGL*-L group treated with 150 mg/kg *FYGL*; *FYGL*-M group treated with 300 mg/kg *FYGL* and *FYGL*-H group treated with 600 mg/kg *FYGL*.

After 7 weeks of drug treatment, all rats were anesthetized with pentobarbital and sacrificed. The abdominal aortas were quickly removed and immersed in 10% formalin for fixation. Then, these tissues were embedded in paraffin and cut into 4 μm sections. The rehydrated sections were stained with hematoxylin-eosin (H&E) for morphology observation [60]. Furthermore, immunohistology was used to evaluate the accumulation of AGEs in the tissues according to the methods of Wang, with modifications [61]. After the activity of endogenous peroxidase was blocked, the aorta sections were incubated with a rabbit anti-AGE antibody (1:1200) overnight at 4 °C, and then the sections were treated by Ultra Sensitive^TM^ SP kit (Maxim Biotechnology Development Co., Ltd., Fuzhou, China) according to the instructions. AGEs in tissue were colored brown by 3,3′-diaminobenzidine (DAB) and observed by NanoZoomer 2.0 HT scanner C12000 (Hamamatsu, Japan). The accumulation of AGEs was quantified by integrated optical density (IOD) using Image-Pro Plus 6.0 software.

### 3.6. Statistical Analysis

All experiments were repeated at least three times, and the results are expressed as mean ± standard deviation (SD). Differences between groups were calculated by one-way ANOVA followed by the Bonferroni post hoc test, and *p* < 0.05 was considered statistically significant.

## 4. Conclusions

This work investigated the effect of *FYGL* on α-glucosidase activity and anti-non-enzymatic glycation in vitro. *FYGL* showed a reversible inhibition for α-glucosidase activity in a mixed model. In addition, fluorescence spectroscopy also showed that the intrinsic fluorescence of α-glucosidase was quenched by *FYGL* due to the formation of a *FYGL*–α-glucosidase complex, resulting in the decrease in enzyme activity. During the anti-non-enzymatic glycation process, *FYGL* inhibited every stage of the glycation process and especially efficiently inhibited the production of AGEs and amyloid fibrillation generated in the end stage, possibly due to its anti-oxidative and scavenging radical capacities to prevent the glycoxidation and crosslinking of glycated BSA. Interestingly, in vivo, *FYGL* alleviated PBG levels demonstrated by OSTT and suppressed the accumulation of AGEs in aortic tissues, thereby reducing vascular injury. Taken together, these results provide support for the development of *FYGL* as an anti-diabetic agent and a novel α-glucosidase and glycation inhibitor against diabetic complications.

## Data Availability

The data presented in this study are available within the article.

## Supplementary Materials

Supplementary materials are available online. Figure S1: ^1^H-NMR spectrum of *FYGL* in D_2_O solution.

## Sample Availability

Samples of *FYGL* are available from the authors.

## References

[1] Joshi S.R., Karne R. (2007). Pre-diabetes, dysglycaemia and early glucose intolerance and vascular health. J. Assoc. Physicians India.

[2] Monnier L., Lapinski H., Colette C. (2003). Contributions of fasting and postprandial plasma glucose increments to the overall diurnal hyperglycemia of type 2 diabetic patients: Variations with increasing levels of HbA(1c). Diabetes Care.

[3] Song P., Onishi A., Koepsell H., Vallon V. (2016). Sodium glucose cotransporter SGLT1 as a therapeutic target in diabetes mellitus. Expert Opin. Ther. Targets.

[4] Joshi S.R., Standl E., Tong N., Shah P., Kalra S., Rathod R. (2015). Therapeutic potential of α-glucosidase inhibitors in type 2 diabetes mellitus: An evidence-based review. Expert Opin. Pharmacother..

[5] Negre-Salvayre A., Salvayre R., Augé N., Pamplona R., Portero-Otín M. (2009). Hyperglycemia and glycation in diabetic complications. Antioxid. Redox Signal..

[6] Goldin A., Beckman J.A., Schmidt A.M., Creager M.A. (2006). Advanced glycation end products - sparking the development of diabetic vascular injury. Circulation.

[7] Rhee S.Y., Kim Y.S. (2018). The role of advanced glycation end products in diabetic vascular complications. Diabetes Metab. J..

[8] Potenza A.M., Gagliardi S., Nacci C., Carratu R.M., Montagnani M. (2009). Endothelial dysfunction in diabetes: From mechanisms to therapeutic targets. Curr. Med. Chem..

[9] Puls W., Kuhlmann J., Puls W. (1996). Pharmacology of glucosidase inhibitors. Oral Antidiabetics.

[10] Bischoff H. (1994). Pharmacology of alpha-glucosidase inhibition. Eur. J. Clin. Investig..

[11] Aldini G., Vistoli G., Stefek M., Chondrogianni N., Grune T., Sereikaite J., Sadowska-Bartosz I., Bartosz G. (2013). Molecular strategies to prevent, inhibit, and degrade advanced glycoxidation and advanced lipoxidation end products. Free Radic. Res..

[12] Luis Ros J., Francini F., Schinella G.R. (2015). Natural products for the treatment of type 2 diabetes mellitus. Planta Med..

[13] Dariya B., Nagaraju G.P. (2020). Advanced glycation end products in diabetes, cancer and phytochemical therapy. Drug Discov. Today.

[14] Bishop K.S., Kao C.H.J., Xu Y., Glucina M.P., Paterson R.R.M., Ferguson L.R. (2015). From 2000 years of *Ganoderma lucidum* to recent developments in nutraceuticals. Phytochemistry.

[15] Hsu K.-D., Cheng K.-C. (2018). From nutraceutical to clinical trial: Frontiers in *Ganoderma* development. Appl. Microbiol. Biotechnol..

[16] Cheng S., Sliva D. (2015). *Ganoderma lucidum* for cancer treatment: We are close but still not there. Integr. Cancer Ther..

[17] Ma H.-T., Hsieh J.-F., Chen S.-T. (2015). Anti-diabetic effects of *Ganoderma lucidum*. Phytochemistry.

[18] Sun L.-X., Lin Z.-B., Lu J., Li W.-D., Niu Y.-D., Sun Y., Hu C.-Y., Zhang G.-Q., Duan X.-S. (2017). The improvement of M_1_ polarization in macrophages by glycopeptide derived from *Ganoderma lucidum*. Immunol. Res..

[19] Kan Y., Chen T., Wu Y., Wu J., Wu J. (2015). Antioxidant activity of polysaccharide extracted from *Ganoderma lucidum* using response surface methodology. Int. J. Biol. Macromol..

[20] Teng B.-S., Wang C.-D., Yang H.-J., Wu J.-S., Zhang D., Zheng M., Fan Z.-H., Pan D., Zhou P. (2011). A protein tyrosine phosphatase 1B activity inhibitor from the fruiting bodies of *Ganoderma lucidum* (Fr.) Karst and its hypoglycemic potency on streptozotocin-induced type 2 diabetic mice. J. Agric. Food Chem..

[21] Pan D., Wang L., Chen C., Hu B., Zhou P. (2015). Isolation and characterization of a hyperbranched proteoglycan from *Ganoderma lucidum* for anti-diabetes. Carbohydr. Polym..

[22] Yu F., Teng Y., Yang S., He Y., Zhang Z., Yang H., Ding C.-F., Zhou P. (2022). The thermodynamic and kinetic mechanisms of a *Ganoderma lucidum* proteoglycan inhibiting hIAPP amyloidosis. Biophys. Chem..

[23] Teng B.S., Wang C.D., Zhang D., Wu J.S., Pan D., Pan L.F., Yang H.J., Zhou P. (2012). Hypoglycemic effect and mechanism of a proteoglycan from *Ganoderma lucidum* on streptozotocin-induced type 2 diabetic rats. Eur. Rev. Med. Pharmacol. Sci..

[24] Singh V.P., Bali A., Singh N., Jaggi A.S. (2014). Advanced glycation end products and diabetic complications. Korean J. Physiol. Pharmacol..

[25] Schalkwijk C.G., Stehouwer C.D.A. (2019). Methylglyoxal, a highly reactive dicarbonyl compound, in diabetes, its vascular complications, and other age-related diseases. Physiol. Rev..

[26] Wang Y., Zhang G., Pan J., Gong D. (2015). Novel insights into the inhibitory mechanism of kaempferol on xanthine oxidase. J. Agric. Food Chem..

[27] Fang Y., Wang S., Wu J., Zhang L., Wang Z., Gan L., He J., Shi H., Hou J. (2017). The kinetics and mechanism of α-glucosidase inhibition by F5-SP, a novel compound derived from sericin peptides. Food Funct..

[28] Huang Q., Chai W.-M., Ma Z.-Y., Ou-Yang C., Wei Q.-M., Song S., Zou Z.-R., Peng Y.-Y. (2019). Inhibition of α-glucosidase activity and non-enzymatic glycation by tannic acid: Inhibitory activity and molecular mechanism. Int. J. Biol. Macromol..

[29] Xiao H., Chen C., Li C., Huang Q., Fu X. (2019). Physicochemical characterization, antioxidant and hypoglycemic activities of selenized polysaccharides from *Sargassum pallidum*. Int. J. Biol. Macromol..

[30] Wang S., Xie X., Zhang L., Hu Y.-M., Wang H., Tu Z.-C. (2020). Inhibition mechanism of α-glucosidase inhibitors screened from *Artemisia selengensis* Turcz root. Ind. Crops Prod..

[31] Tian G.C., Sobotka-Briner C.D., Zysk J., Liu X.D., Birr C., Sylvester M.A., Edwards P.D., Scott C.D., Greenberg B.D. (2002). Linear non-competitive inhibition of solubilized human gamma-secretase by pepstatin a methylester, L685458, sulfonamides, and benzodiazepines. J. Biol. Chem..

[32] Peng X., Zhang G., Liao Y., Gong D. (2016). Inhibitory kinetics and mechanism of kaempferol on α-glucosidase. Food Chem..

[33] Xiao H.Z., Liu B.G., Mo H.Z., Liang G.Z. (2015). Comparative evaluation of tannic acid inhibiting alpha-glucosidase and trypsin. Food Res. Int..

[34] Liu M.C., Jin S.F., Zheng M., Wang Y., Zhao P.L., Tang D.T., Chen J., Lin J.Q., Wang X.H., Zhao P. (2016). Daunomycin-loaded superparamagnetic iron oxide nanoparticles: Preparation, magnetic targeting, cell cytotoxicity, and protein delivery research. J. Biomater. Appl..

[35] Xie F., Gong S., Zhang W., Wu J., Wang Z. (2017). Potential of lignin from *Canna edulis* ker residue in the inhibition of α-D-glucosidase: Kinetics and interaction mechanism merging with docking simulation. Int. J. Biol. Macromol..

[36] Zhang G., Wang L., Pan J. (2012). Probing the binding of the flavonoid diosmetin to human serum albumin by multispectroscopic techniques. J. Agric. Food Chem..

[37] Han L., Fang C., Zhu R., Peng Q., Li D., Wang M. (2017). Inhibitory effect of phloretin on α-glucosidase: Kinetics, interaction mechanism and molecular docking. Int. J. Biol. Macromol..

[38] Yang B., Wang J., Zhao M., Liu Y., Wang W., Jiang Y. (2006). Identification of polysaccharides from pericarp tissues of litchi (*Litchi chinensis* Sonn.) fruit in relation to their antioxidant activities. Carbohydr. Res..

[39] Chen H., Zhang M., Qu Z., Xie B. (2008). Antioxidant activities of different fractions of polysaccharide conjugates from green tea (*Camellia sinensis*). Food Chem..

[40] Zeng L., Ding H.F., Hu X., Zhang G.W., Gong D.M. (2019). Galangin inhibits alpha-glucosidase activity and formation of non-enzymatic glycation products. Food Chem..

[41] Johnson R.N., Metcalf P.A., Baker J.R. (1983). Fructosamine: A new approach to the estimation of serum glycosylprotein. An index of diabetic control. Clin. Chim. Acta.

[42] Wells-Knecht K.J., Zyzak D.V., Litchfield J.E., Thorpe S.R., Baynes J.W. (1995). Identification of glyoxal and arabinose as intermediates in the autoxidative modification of proteins by glucose. Biochemistry.

[43] Thornalley P.J., Rabbani N. (2014). Detection of oxidized and glycated proteins in clinical samples using mass spectrometry—A user’s perspective. Biochimica et Biophys. Acta (BBA)-Gen. Subj..

[44] Ding H., Ni M., Zhang G., Liao Y., Hu X., Zhang Y., Gong D. (2020). The inhibition of oleanolic acid on protein non-enzymatic glycation. LWT.

[45] Taghavi F., Habibi-Rezaei M., Amani M., Saboury A.A., Moosavi-Movahedi A.A. (2017). The status of glycation in protein aggregation. Int. J. Biol. Macromol..

[46] Sadowska-Bartosz I., Galiniak S., Bartosz G. (2014). Kinetics of glycoxidation of bovine serum albumin by methylglyoxal and glyoxal and its prevention by various compounds. Molecules.

[47] Zhang Q., Huang Z., Wang Y., Wang Y., Fu L., Su L. (2021). Chinese bayberry (*Myrica rubra*) phenolics mitigated protein glycoxidation and formation of advanced glycation end-products: A mechanistic investigation. Food Chem..

[48] Siriamornpun S., Kaewseejan N., Chumroenphat T., Inchuen S. (2021). Characterization of polysaccharides from *Gynura procumbens* with relation to their antioxidant and anti-glycation potentials. Biocatal. Agric. Biotechnol..

[49] Zhang L., Xu L., Tu Z.-C., Wang H.-H., Luo J., Ma T.-X. (2020). Mechanisms of isoquercitrin attenuates ovalbumin glycation: Investigation by spectroscopy, spectrometry and molecular docking. Food Chem..

[50] Wu X., Zhang G., Hu M., Pan J., Li A., Zhang Y. (2020). Molecular characteristics of gallocatechin gallate affecting protein glycation. Food Hydrocoll..

[51] Pan D., Zhang D., Wu J., Chen C., Xu Z., Yang H., Zhou P. (2013). Antidiabetic, antihyperlipidemic and antioxidant activities of a novel proteoglycan from *Ganoderma lucidum* fruiting bodies on *db/db* mice and the possible mechanism. PLoS ONE.

[52] Xu B., Ji Y., Yao K., Cao Y.X., Ferro A. (2005). Inhibition of human endothelial cell nitric oxide synthesis by advanced glycation end-products but not glucose: Relevance to diabetes. Clin. Sci..

[53] Liu M., Yin H., Liu G., Dong J., Qian Z., Miao J. (2014). Xanthohumol, a prenylated chalcone from beer hops, acts as an α-glucosidase inhibitor in vitro. J. Agric. Food Chem..

[54] Wang S.-H., Chang J.-C., Pokkaew R., Lee J.-F., Chiou R.Y.Y. (2011). Modified fast procedure for the detection and screening of antiglycative phytochemicals. J. Agric. Food Chem..

[55] Sadowska-Bartosz I., Galiniak S., Bartosz G. (2014). Kinetics of glycoxidation of bovine serum albumin by glucose, fructose and ribose and its prevention by food components. Molecules.

[56] Bouma B., Kroon-Batenburg L.M.J., Wu Y.-P., Brünjes B., Posthuma G., Kranenburg O., de Groot P.G., Voest E.E., Gebbink M.F.B.G. (2003). Glycation induces formation of amyloid cross-β structure in albumin. J. Biol. Chem..

[57] Eze F.N., Leelawatwattana L., Prapunpoj P. (2019). Structural stabilization of human transthyretin by *Centella asiatica* (L.) urban extract: Implications for TTR amyloidosis. Biomolecules.

[58] Franco R.R., Ribeiro Zabisky L.F., de Lima Júnior J.P., Mota Alves V.H., Justino A.B., Saraiva A.L., Goulart L.R., Espindola F.S. (2020). Antidiabetic effects of *Syzygium cumini* leaves: A non-hemolytic plant with potential against process of oxidation, glycation, inflammation and digestive enzymes catalysis. J. Ethnopharmacol..

[59] Li T., Wang L., Chen Z., Zhang X., Zhu Z. (2020). Functional properties and structural changes of rice proteins with anthocyanins complexation. Food Chem..

[60] Wang Z., Zhang J., Chen L., Li J., Zhang H., Guo X. (2019). Glycine suppresses AGE/RAGE signaling pathway and subsequent oxidative stress by restoring Glo1 function in the aorta of diabetic rats and in HUVECs. Oxid. Med. Cell. Longev..

[61] Wang Z., Zhang J., Wang L., Li W., Chen L., Li J., Zhao D., Zhang H., Guo X. (2018). Glycine mitigates renal oxidative stress by suppressing Nox4 expression in rats with streptozotocin-induced diabetes. J. Pharmacol. Sci..

