# Supplementary material for: Inhibition on α-Glucosidase Activity and Non-Enzymatic Glycation by an Anti-Oxidative Proteoglycan from Ganoderma lucidum
Source: Molecules. 2022 Feb 22;27(5):1457. doi: 10.3390/molecules27051457 (PMC8912016; doi:10.3390/molecules27051457)
Supplement: Supplementary file 1 [file molecules-27-01457-s001.zip › molecules-1594178-supplementary.pdf]

Inhibition on α-Glucosidase Activity and Non-Enzymatic Glycation by an Anti-Oxidative Proteoglycan from *Ganoderma lucidum*

Ying Zhang [1], Yanna Pan [1], Jiaqi Li [1], Zeng Zhang [2], Yanming He [2], Hongjie Yang [2,*] and Ping Zhou [1,*]

[1] State Key Laboratory of Molecular Engineering of Polymers, Department of Macromolecular Science, Fudan University, Shanghai 200433, China; 19210440005@fudan.edu.cn (Y.Z.); 18110440051@fudan.edu.cn (Y.P.); 20210440013@fudan.edu.cn (J.L.)

[2] Yueyang Hospital of Integrated Traditional Chinese and Western Medicine, Shanghai University of Traditional Chinese Medicine, Shanghai 200437, China; zengzeng31@163.com (Z.Z.); heyanming176@163.com (Y.H.)

* Correspondence: yanghongjie1964@aliyun.com (H.Y.); pingzhou@fudan.edu.cn (P.Z.); Tel./Fax: +86-21-3124-4038 (P.Z.)

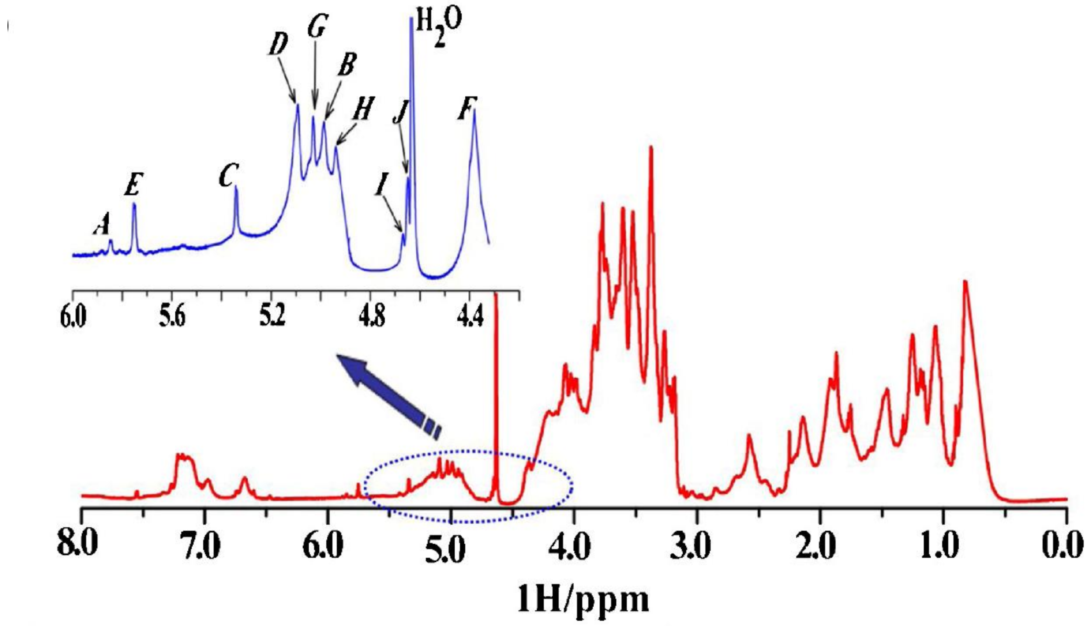

S1

Figure S1. ^{1}H-NMR spectrum of *FYGL* in D_2O solution[1].

References

1. Pan, D.; Wang, L.; Chen, C.; Hu, B.; Zhou, P. Isolation and characterization of a hyperbranched proteoglycan from *Ganoderma lucidum* for anti-diabetes. *Carbohydr. Polym.* **2015**, *117*, 106-114 (10.1016/j.carbpol.2014.09.051).
